# A hybrid zone between *Bathymodiolus* mussel lineages from eastern Pacific hydrothermal vents

**DOI:** 10.1186/1471-2148-13-21

**Published:** 2013-01-24

**Authors:** Shannon B Johnson, Yong-Jin Won, Julio BJ Harvey, Robert C Vrijenhoek

**Affiliations:** 1 Monterey Bay Aquarium Research Institute, Moss Landing, CA, 95039-9644, USA; 2 Division of EcoScience, Ewha Womans University, Seoul, 120-750, South Korea

**Keywords:** *Bathymodiolus antarcticus* n. sp, Hybridization, Recombination, Linkage disequilibrium, Deep-sea, Hydrothermal vent, *Bathymodiolus thermophilus*

## Abstract

**Background:**

The inhabitants of deep-sea hydrothermal vents occupy ephemeral island-like habitats distributed sporadically along tectonic spreading-centers, back-arc basins, and volcanically active seamounts. The majority of vent taxa undergo a pelagic larval phase, and thus varying degrees of geographical subdivision, ranging from no impedance of dispersal to complete isolation, often exist among taxa that span common geomorphological boundaries. Two lineages of *Bathymodiolus* mussels segregate on either side of the Easter Microplate, a boundary that separates the East Pacific Rise from spreading centers connected to the Pacific-Antarctic Ridge.

**Results:**

A recent sample from the northwest flank of the Easter Microplate contained an admixture of northern and southern mitochondrial haplotypes and corresponding alleles at five nuclear gene loci. Genotypic frequencies in this sample did not fit random mating expectation. Significant heterozygote deficiencies at nuclear loci and gametic disequilibria between loci suggested that this transitional region might be a ‘Tension Zone’ maintained by immigration of parental types and possibly hybrid unfitness. An analysis of recombination history in the nuclear genes suggests a prolonged history of parapatric contact between the two mussel lineages. We hereby elevate the southern lineage to species status as *Bathymodiolus antarcticus* n. sp. and restrict the use of *Bathymodiolus thermophilus* to the northern lineage.

**Conclusions:**

Because *B. thermophilus* s.s. exhibits no evidence for subdivision or isolation-by-distance across its 4000 km range along the EPR axis and Galápagos Rift, partial isolation of *B. antarcticus* n. sp. requires explanation. The time needed to produce the observed degree of mitochondrial differentiation is consistent with the age of the Easter Microplate (2.5 to 5.3 million years). The complex geomorphology of the Easter Microplate region forces strong cross-axis currents that might disrupt self-recruitment of mussels by removing planktotrophic larvae from the ridge axis. Furthermore, frequent local extinction events in this tectonically dynamic region might produce a demographic sink rather than a source for dispersing mussel larvae. Historical changes in tectonic rates and current patterns appear to permit intermittent contact and introgression between the two species.

## Background

Hybrid zones, regions of intergradation between genetically distinct populations or species, have been documented for a number of terrestrial and aquatic organisms. The locations, shapes and ages of these zones, and the unique dispersal, behavioral and fitness characteristics of the participating organisms have motivated a number of models that invoke endogenous (i.e. genetic) or exogenous (i.e. environmental) forces reviewed by [1-5]. Intergradation can result from primary or secondary processes that are difficult to distinguish [6]. Primary intergradation, which occurs at the contact boundaries between distinct habitats or along steep environmental gradients, is associated with parapatric speciation. Allelic and phenotypic frequencies often exhibit step-clines shaped by disruptive selection across the contact zone. Secondary intergradation, resulting from contact between previously allopatric populations, can produce similar step-clines. Different histories of dispersal and selection can produce similar patterns of concordance in allelic frequency clines at multiple gene loci, though subtle and discriminating footprints may remain in the genetic architecture of intergrading lineages [1].

**Figure 1:**
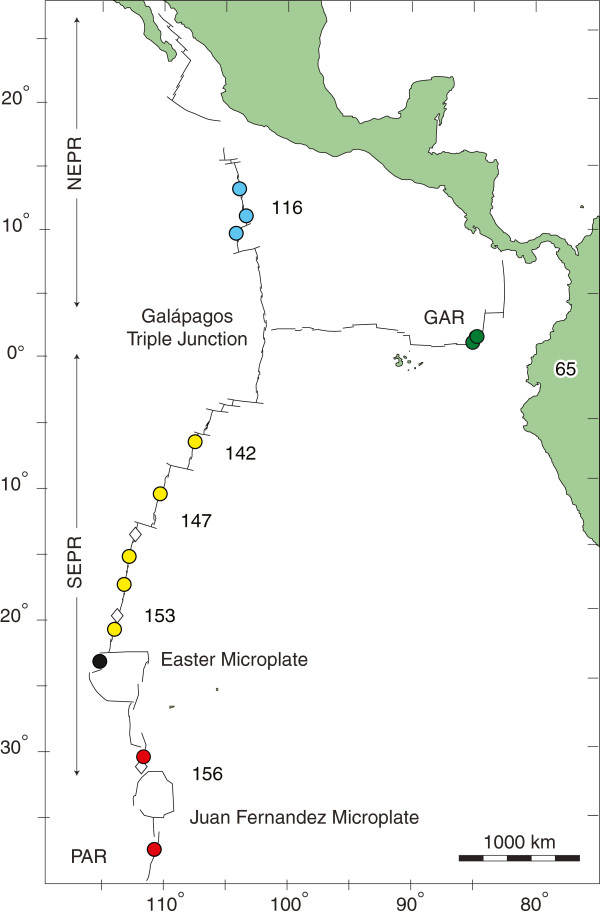
**Sample localities for eastern Pacific *Bathymodiolus *are marked by a color filled circle in accordance with the following regions: NEPR denotes the northern East Pacific Rise; SEPR = southern East Pacific Rise; GAR = Galápagos Rift; and PAR = Pacific-Antarctic Ridge.** Numbers indicate spreading rates of ridge segments in mm/year.

Most examples of hybrid zones come from terrestrial and freshwater environments that have experienced past climatic changes — for example, at suture zones where formerly isolated biotic assemblages attain secondary contact [7,8]. Hybrid zones are uncommon in the open ocean, because marine environments tend to be more homogeneous temporally and spatially, and many marine species have high dispersal capabilities [9]. Nonetheless, marine hybrid zones are found in coastal environments that experience steep environmental gradients or were impacted by past climatic changes [10]. Hybridization is prevalent among subtidal mussels, *Mytilus edulis, M. galloprovincialis,* and *M. trossulus*, members of the “blue mussel complex” [11,12]. These closely related species are known to readily hybridize wherever two blue mussels are sympatric [13-15]. A mid-ocean hybrid zone was first discovered in deep-sea hydrothermal vent mussels (Mollusca: Bivalvia: Mytilidae) living along the Mid-Atlantic Ridge [16]. Hydrothermal vents differ from other open oceanic habitats because they occur as discrete island-like habitats distributed along the global mid-ocean ridge system (Figure 1), in disjunct back-arc spreading centers, and on active seamounts [17]. Because vent communities rely almost entirely on reduced volcanic gases (primarily H_2_S and CH_4_,) and chemosynthetic bacteria for primary productivity, habitat quality can vary greatly in time and space [18]. The larvae of most vent animals are dispersed by hydrographic circulation that can be disrupted by geomorphological structures, such as ridge offsets (e.g., transform faults), intervening seamounts, and other bathymetric features [19,20]. Consequently, genetic studies have often revealed geographic subdivision and species boundaries associated with such features [21,22].

The Mid-Atlantic Ridge (MAR) mussels, *Bathymodiolus azoricus* and *B. puteoserpentis*, mussels segregate latitudinally and hybridize at an intermediate location that is relatively inhospitable for mussels [16,23]. The hybrid population exhibits an excess of the parental cytonuclear genotypes, leading Won *et al.*[24] to hypothesize that the contact region is Tension Zone [sensu 1] maintained primarily by immigration from the parental zones and possibly hybrid unfitness. A coalescence analysis of the nuclear *GF1B* locus identified recombination events between branch tips belonging to the parental lineages [25]. The degree of mitochondrial divergence and the apparent absence of earlier inter-lineage recombination events at *GF1B* were interpreted as evidence for a period of allopatric differentiation beginning about 0.760 million years (Myr) ago, followed by prolonged secondary contact and introgression.

Discovery of the MAR hybrid zone stimulated our interests in other potential contact zones along the global mid-ocean ridge system. Our target was the Easter Microplate boundary (EMB, Figure 1), where vent fauna associated with the 7000-km long East Pacific Rise (EPR) contact a related fauna associated with a NE segment of the Pacific-Antarctic Ridge (PAR) [22]. The EMB separates geminate species of bythograeid crabs [26,27], lepetodrilid limpets [28], and *Bathymodiolus* mussels [29]. The EMB does not contribute to significant geographical partitioning of all lepetodrilid species [28] and annelid species [30,31], however. Instead, this region acts as a “variable dispersal filter” with affects that depend on the life history characteristics and dispersal modes of individual taxa [22]. To better define this contact zone, we explored and sampled vents that were previously reported to exist in the region between 23°S on the NW flank of the Easter Microplate and 38°S latitude along the PAR [32,33].

Herein, we report the discovery of a zone of intergradation between two mussel lineages that contact one another at the EMB. *Bathymodiolus thermophilus* Kenk & Wilson [34] is distributed on the Galápagos Rift (GAR) and East Pacific Rise (EPR) between 13°N and 21°S latitude. A related lineage, *Bathymodiolus* aff. *thermophilus*[29], was found at 31–32°S latitude on segments of the EPR axis connecting the Easter and the Juan de Fernandez microplates. The mussel lineages are reciprocally monophyletic for distinct mitochondrial *COI* haplotypes that are 4.6% divergent, and they exhibit significant shifts in allozyme frequencies at five polymorphic loci. These concordant differences at multiple, independent, gene loci led Won *et al.*[29] to suggest that the southern lineage might warrant consideration as a distinct species, but additional samples and analyses of independent nuclear gene loci were needed to assess this hypothesis. With these goals, we examined mitochondrial *COI* and portions of five nuclear genes from mussels sampled along a 5600-km range from 13°N to 38°S latitude (Figure 1). Multilocus genotypes constructed from the DNA sequences were used to assess geographical structure and subdivision, to identify putative hybrids, and to test for gametic disequilibrium and recombination in the contact zone.

## Methods

### Samples

Expeditions conducted between 1990 and 2005 explored segments of the GAR, EPR and PAR (Figure 1) to obtain specimens for multi-species phylogeographic studies reviewed in [22]. Exploration in the Easter Microplate region was guided by shipboard coordinates and towed-video surveys reported in a PhD thesis [35]. Mussel samples were obtained from 14 localities, subsequently labeled with abbreviations that include the approximate latitude, e.g., N13, N11, … S31, S38 (Table 1). Samples collected with the human-occupied vehicle (HOV) *Alvin* were placed in an insulated ‘biobox’ containing ambient seawater at 2–4°C. Adult mussels (up to ~15 cm in length) were sampled with scoops, nets or directly with *Alvin*’s robotic manipulators. The S23 sample of juveniles (≤25 mm) was attached by byssal threads to a rock sampled for geological studies. Upon recovery of the submersible, all samples were stored temporarily in refrigerated seawater prior to dissection of gill and adductor muscle tissues that were frozen at −80°C or preserved in 95% ethanol.

**Table 1:** **Sample coordinates for eastern Pacific *Bathymodiolus *** **mussels and sample sizes examined for six gene loci**

**Sample**	**Decimal coordinates**		**Depth**				**Number of individuals sampled**					
**No.**	**Lat.**	**Long.**	**range (m)**	**Year**	**Dive nos.**^**1**^	**Status**^**2**^	***COI***^**3**^	***SAHH***	***ANT***	***Cat***	***Col-1***	***Ef1α***
N13	12.80	−103.93	2636	1990	A2228/29	L	18(12)	16	13	18	16	16
N11	11.4	−103.78	2516	1990	A2225/26	L	15(12)	15	14	13	14	14
N9	9.55/80	−104.25/28	2567	1991	A2350/52	L	20(12)	13	17	13	12	11
	9.52	−104.25	2500/2585	1999	H	45						
GAR	0.80	−86.15/22	2461/2515	1990	A2223/24	L	35(24)	14	26	25	12	11
S7	−7.37/42	−107.78/80	2746/7	1999	A3320	L	12(12)	11	12	11	10	9
	−7.37	−107.78	2746	2004	B	27						
S11	−11.30	−110.53	2665	1999	A3323	L	15(12)	10	11	10	11	11
S14	−13.98	−112.48	2625/2627	1999	A3324/25	S						
			2623/2632	2004	B	14						
S17	−17.40/53	−113.20/23	2578	1999	A3327/30	L	31(12)	19	19	21	20	9
	−17.40	−113.25	2595	2004	B	87						
S18	−18.40/43	−113.38/40	2628	1999	A3331/33	S						
	−18.43	−113.40	2636/2680	2004	B	47						
S20	−20.05	−113.68	2804/2840	1999	A3334	S						
S21	−21.57	−114.30	2830/2836	1999	A3335/36	no						
	−21.42	−113.40	2804/2840	2004	B	34						
S23	−23.53	−115.57	2595	2005	A4096	J, S	21	21	20	20	21	21
S31	−31.15	−111.93	2237	1999	A3337/39	L	(12)					
	−31.15	−111.93	2237	2005	A4094	L	26	23	27	28	23	25
S32	−31.83/85	−111.92/07	2331	1999	A3340/42	L						
	−31.85	−112.03	2332	2005	A4092/3	L						
S38	−37.78/79	−110.90/91	2215/2233	2005	A4087/91	L	27	29	26	33	27	30

^1^ Dive numbers preceded by letter “A” are HOV *Alvin* dives. H = HOPE expedition, and B = BIOSPEEDO expeditions see [36].

^2^ Status: numbers = sample sizes from Plouviez *et al.* 2009 (GenBank acc. nos. GQ473649–902); L = living adult mussels observed; S = shell debris observed; J = juveniles sampled with a sulfide rock; and “no” = not observed.

^3^ In parentheses are sample sizes from Won *et al.* 2003 (GenBank acc. nos. AF456282–322).

## DNA methods

DNA extraction and purification, general PCR conditions, amplicon purification and DNA sequencing used methods that were previously reported for mussels [37]. Six primer sets were used to amplify DNA targets from mussels (Table 2). All PCR products were diluted in 40 μl of sterile water and purified with a Multiscreen HTS PCR 96 vacuum manifold system (Millipore Corp. Billerica, MA). PCR products were sequenced bi-directionally on an ABI3130 sequencer with BigDye Terminator v3.1 (Life Technologies Corp., Carlsbad, CA) chemistry and primers used in PCR.

**Table 2:** **PCR primers and methods for six gene loci in *Bathymodiolus *** **mussels**

**Locus**	**Product**	**Primers**	**Ref.**^**1**^	**Methods**	**Length**
*COI*	Cytochrome-*c*-oxidase subunit-I	COIG/H	[37]	^2^	570
*SAHH*	S-adenosylhomocysteine hydrolase	BatSAHHF/370R	[25]^3^	Fast PCR	~400
*ANT*	Adenine nucleotide (ADP/ATP) translocase	AntF/AntR1 and AntF/AntR2	[38]	TD and fast PCR	496
*Cat*	Catchin	CatF/Cat2R	^4^	Fast PCR^5^	~500
*Col-1*	Collagen type XIV	Col160F/Col619R	^6^	Fast PCR	~430
*EF1α*	Elongation Factor 1α	BatEf1αF/BatEf1αR	[25]	Fast PCR	540

^1^ References in Literature Cited.

^2^ PCR program: 95°C/10 min; 35 x [94°C/1 min, 55°C/1 min; 72°C/2 min], extension at 72°C/7 min.

^3^ New to this study: Based on [25], SAHH370R, 5'-CGYTCGAACTCGCAAYGTYAGTGG-3'.

^4^ New to this study: CatF, 5'-GAGYGTCTTTCMAAGATCATCTCC-3'/Cat2R, 5'-CATTTYCTGATGTTWCGCTGGAT-3'.

^5^ Touchdown and Fast PCR: Amplitaq Gold Fast PCR Master Mix, UP (Life Technologies Corp., Carlsbad, CA) and the protocol for the *taq* supplied by manufacturer of Veriti thermal cycler with an annealing temperature at 50°C (Life Technologies Corp., Carlsbad, CA).

^6^ New to this study: Based on [25], Col160F, 5'-GGTTCACGAYCGGAWGTTCCCC-3', Col619R, 5'-TCCCAGCCTTTTCTCGCCCA-3'.

### Molecular statistics

Computer programs used to format the data, obtain estimates of population genetic parameters, and conduct statistical tests are listed along with procedural references (Table 3). We used Phase v. 2.1.1 to ascertain alleles in individuals heterozygous for nuclear genes. Characterization of alleles in individuals heterozygous for insertions and deletions (indels) required cloning and sequencing of at least five clones per individual methods in [39]. Analyses of genic diversity, Hardy-Weinberg equilibrium, gametic disequilibrium, Isolation-with-Migration (IMa2), and Isolation-by-Distance (IBD) were limited to the exon portions of four nuclear genes that contained introns. None of the exon regions in these sequences exhibited stop-codons. The states of a 9-bp indel in the *EF1α* intron were treated as two-alleles. Tests for intra-genic recombination were conducted with Phase v. 2.1.1. Phylogenetic analyses of historical recombination within nuclear genes were conducted with Beagle on complete sequences, including introns and exons.

**Table 3:** Programs used to estimate genetic parameters and conduct statistical tests and procedures

**Program**	**Version**	**Parameters**^**1**^**, tests and embedded procedures**	**References**
Arlequin	3.5.1.3	Diversity indices (*H*, *k*, *h*, π, *F*_*ST*_, φ_*ST*_)	[40]
		Fu’s *Fs* test	[41]
		Linkage disequilibrium (*D*, *D’*, *R*^2^)	[42]
		IBD: Pairwise Φ_*ST*_, *F*_*ST*_ and	[43]
		Mantel test	[44]
Beagle		Recombination test	[45]
Beast	1.7.2	Estimation of divergence time	[46]
CNDm		Cytonuclear disequilibrium (*D*, *D’*)	[47]
CodonCode Aligner	3.7.1.1	Proofreading sequences	CodonCode Corp., Dedham, MA
Genepop	4.0.10	Multilocus data management	[48]
		HWE exact tests	[49]
IMa2	8.26.11	Demographic parameters (*τ*, θ_A_, θ_1_, θ_2_, *m*_1_, *m*_2_)	[50]
Geneious	5.6.2	Sequence editing	[51]
Network Publisher	1.3.0.0	Illustrating gene networks	[52]
NewHybrids	1	Assignment of putative hybrids	[53]
Phase	2.1.1	Decomposing heterozygotes, recombination test	[54,55]
Structure	2.3.3	Assignment test (*K*)	[56,57]

^1^ Parameters: *H*, number of haplotypes; *k*, number of polymorphic sites; *h*, haplotype diversity; *π*, nucleotide diversity per site; *F*_*ST*_, standardized molecular variance among populations; φ_*ST*_, standardized molecular variance among populations; *D*, nuclear linkage disequilibrium (LD); *D’*, standardized LD; *R*^2^, correlation measure of LD; estimators of nuclear linkage disequilibrium; τ, time to most recent common ancestor; θ_A_, θ_1_, θ_2_, sizes of ancestral and descendant populations; *m*_1_, *m*_2_, immigration into descendant populations; *K*, number of genotypic clusters.

Gene networks for the exon portions of each locus were constructed with Network Publisher v. 1.3.0.0. Inferred haplotypes were re-labeled as discrete alleles (*a*^1^, *a*^2^, *…*, *a*^*n*^) and concatenated manually into a multilocus genotype (MLG) file (*a*^1^/*a*^3^, *b*^2^/*b*^2^, *…*, *g*^*1*^*/g*^*1*^). Uninformative singleton alleles were lumped with their most closely related haplotype. The MLG file was used as an input file for Genepop v. 4.0.10. Exact tests for Hardy-Weinberg equilibrium (HWE) were conducted according to the method of Raymond and Rousset [49]. The MLG file was also used as an input file for Arlequin v. 3.5.1.3 to conduct pairwise likelihood ratio tests for gametic disequilibrium and Mantel tests for Isolation-by-Distance (IBD). Sequential Bonferroni corrections were used to adjust α-levels [58].

The MLG file was also used to conduct assignment tests. Structure v. 2.3.3 analyses were conducted with and without prior information about population samples and admixture, and with correlated and uncorrelated allelic frequencies. The first 10^3^ generations (burn-in) were discarded and 10^7^ generations were iterated for 3 to10 times per estimate of *K*, the number of genotypic clusters. Assuming a uniform prior on *K*, we estimated a Bayes factor for each value of *K* after averaging the *ln* Pr(X/*K*) values between simulations. NewHybrids was used to assign the multilocus genotypes to putative parental, F1, F2, or backcross categories. We used default genotypic classes with no prior information on allelic frequencies and included uniform and Jefferey’s priors [59] for *θ* and *π*. Five separate analyses were conducted with 10^5^ sweeps of burn-in and 10^7^ sweeps of data collection. Threshold values (*Tq*) of 0.9 [56] and 0.75 [60] were assigned separately for the genotypic categories if *q ≥ Tq*, and they were left unassigned if *q < Tq*[61].

We used the Isolation-with-Migration method, IMa2[50,62,63] to estimate the following demographic parameters: (*τ*) the time of population splitting; (*θ*_A_, *θ*_N_, *θ*_S_) the sizes of ancestral and descendant populations; and (*m*_N_, *m*_S_) immigration into the descendant populations. An *HKY* substitution model with back mutation was assumed. An inheritance scalar to adjust the relative expected effective population size was 0.25 for mitochondrial *COI* and 1.0 for nuclear loci. Analyses were conducted under a two-population model based on Structure results. The two-population model lumped the N13–S23 and GAR samples into a northern group, and the S31–S38 samples into a southern group. The S23 sample was treated as northern due to the preponderance of northern alleles. A second set of analyses was also run without the S23 population. Analyses involved at least 10^8^ steps with the first 10^4^ steps discarded as burn-in (−b) [64], with 50 attempts at chain swapping per step (−k), 50–80 chains (−n) with geometric heating (−f), and *g1* and *g2* values of 0.99 and 0.3, respectively. Analyses were conducted multiple times and the number of steps between saving genealogies (−d) was increased to 1000. Initially we set upper bounds for each parameter, as recommended in the IMa2 manual, then performed multiple runs using random number seeds with more appropriate bounds based on the initial output. We assessed the convergence of MCMC samplings of population parameters in multiple ways by allowing analyses to run ensuring effective sample sizes were a minimum of 100 for all parameters, examining output graphs for unimodality, ensuring genealogies were updating, and checking that multiple runs gave similar estimates. Multiple runs based on the same priors were then combined in L-mode and log-likelihood ratio tests were performed to test for isolation.

## Results

Adult mussels were abundant at most of the active vent fields between 13°N and 38°S latitude (Figure 1; Table 1). The S23 locality on the NW flank of the Easter Microplate was an exception. During three dive-days (bottom-time = 14.1 hours), we observed a small vent field littered with the shells of dead bivalves. Post-expedition inspection of the dive videos identified one living adult that was not sampled; however, we inadvertently collected 21 juvenile mussels (≤ 25 mm length) that were attached to the underside of a sulfide rock sampled for geological studies. The S23 site, located in a deep basaltic crevice, was recently more active. Sulfide rubble from dead chimneys was scattered about, but we found only one active vent chimney with a high temperature of 296°C. The dominant animals were typical of the fauna found at the periphery of active vents — e.g., serpulid annelids, caridean shrimp, anemones, enteropneusts, crinoids, brisingid sea stars, holothurians, sponges, and siphonophores [65]. Typical vent fauna, like alvinellid polychaetes were observed but not abundant. Two living *Riftia pachyptila* tubeworms were observed and one was collected [31]. Remnant tubes of *Tevnia jerichonana* occurred on sulfide rubble covering an area of diffuse low-temperature (≤ 8.5°C) venting, but no living worms were collected. Overall, the S23 locality exhibited hallmarks of a senescent vent field.

### Geographical subdivision and admixture

**Figure 2:**
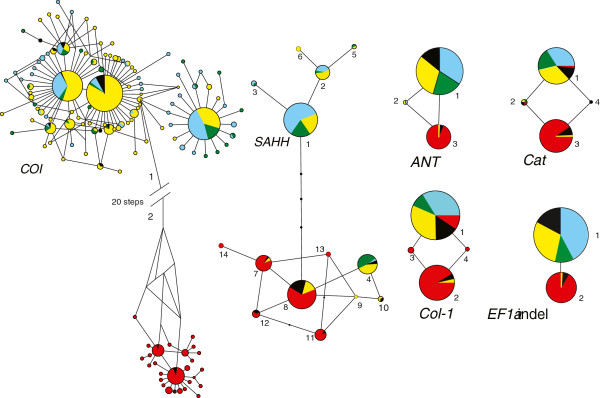
**Gene networks for six loci in eastern Pacific *Bathymodiolus.*** Numbers represent alleles used in the statistical analyses and black dots represent missing haplotypes. Colored pie slices denote regions, as indicated in Figure 1: NEPR = blue; GAR = green; SEPR = yellow; S23 = black S23; and PAR = red.

**Figure 3:**
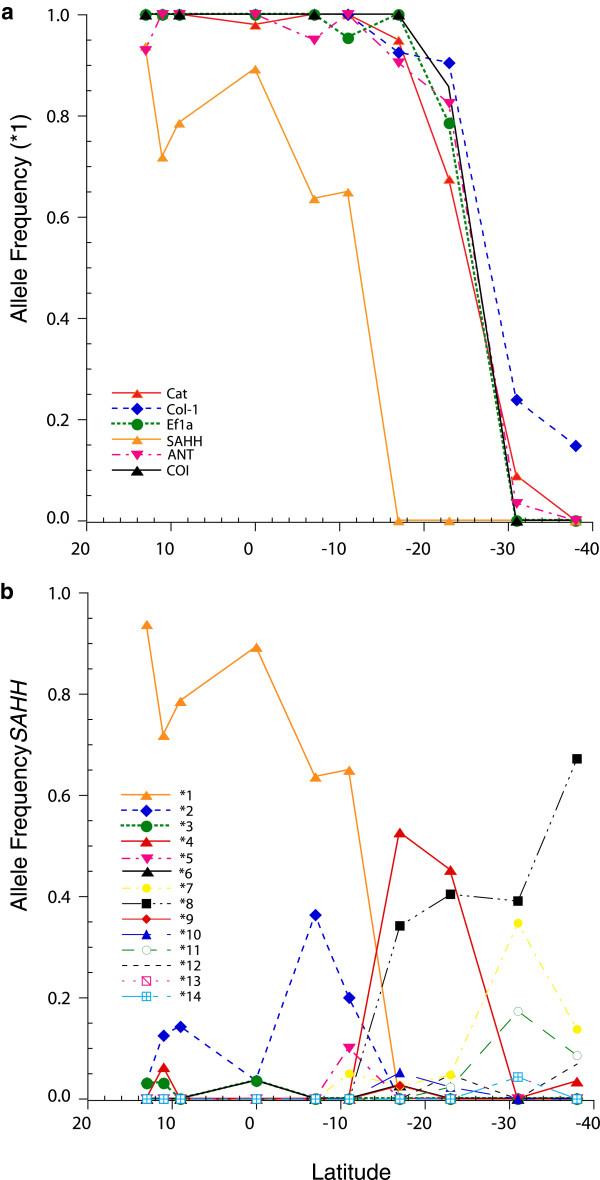
**Latitudinal clines.(a)** Step-clines for the predominant northern allele (haplotype: *1) at six loci toward southern direction from left to right **(b)** Allelic frequencies of *SAHH* alleles.

Analyses were conducted with sequence polymorphisms in mitochondrial *COI* and the exon portions of four nuclear genes, *SAHH*, *ANT*, *Cat* and *Col-1* (Table 4). A 9-bp indel in *EF1α* intron was treated as two discrete alleles. Haplotype networks for all six loci revealed differentiation between northern (*N*) and southern (*S*) mussel lineages (Figure 2). Distinct *N* and *S* haplotypes for *COI* were reciprocally monophyletic and separated by at least 20 substitutions. Though less divergent, predominantly *N* and *S* alleles segregated at all five nuclear loci (Figure 2). For each locus, the S23 sample (black pie slices, Figure 2) included *N* and *S* alleles. Latitudinal step-clines for five loci had common inflection points centered on the S23 locality (Figure 3a). The *SAHH* cline was more complex (Figure 3b). Frequencies of the northern alleles, SAHH*1 and *2, declined to zero at S17, counterbalanced by increases of SAHH*7 and *8. The SAHH*4 allele only occurred in the intermediate region, S17 and S23. 

**Table 4:** **Gene characteristics and GenBank accession numbers for six gene loci in eastern Pacific *Bathymodiolus *** **mussels**

**Locus**	**Length**	**Exon bp**	***S***^**1**^	**H**^**2**^	**R**_**e**_^**3**^	**Intron bp**	**Indels**^**4**^	**R**_**i**_^**3**^	**GenBank acc. Nos.**
*COI*	570	1–570	96	121	no	N/A	N/A	N/A	JN978437-JN987653
*SAHH*	225	1–159	9	14	no	160-225	10 bp	yes	JX890706-JX891047
*ANT*	496	1-496	1	3	no	N/A	no	no	JN978654-JN979017
*Cat*	350	1–38	2	4	no	39-350	no	yes	JN979018-JN979369
*Col-1*	426	1–104	3	4	no	105–426	no	yes	JX891048-JX891379
*EF1α*	456	1-100	0	0	N/A	101-456	9 bp	yes	JX890392-JX890705

^1^ Polymorphic sites.

^2^ Number of haplotypes (alleles).

^3^ Tests for recombination in exon (*R*_*e*_) and intron (*R*_*i*_) regions.

^4^ Insertions and deletions.

**Figure 4:**
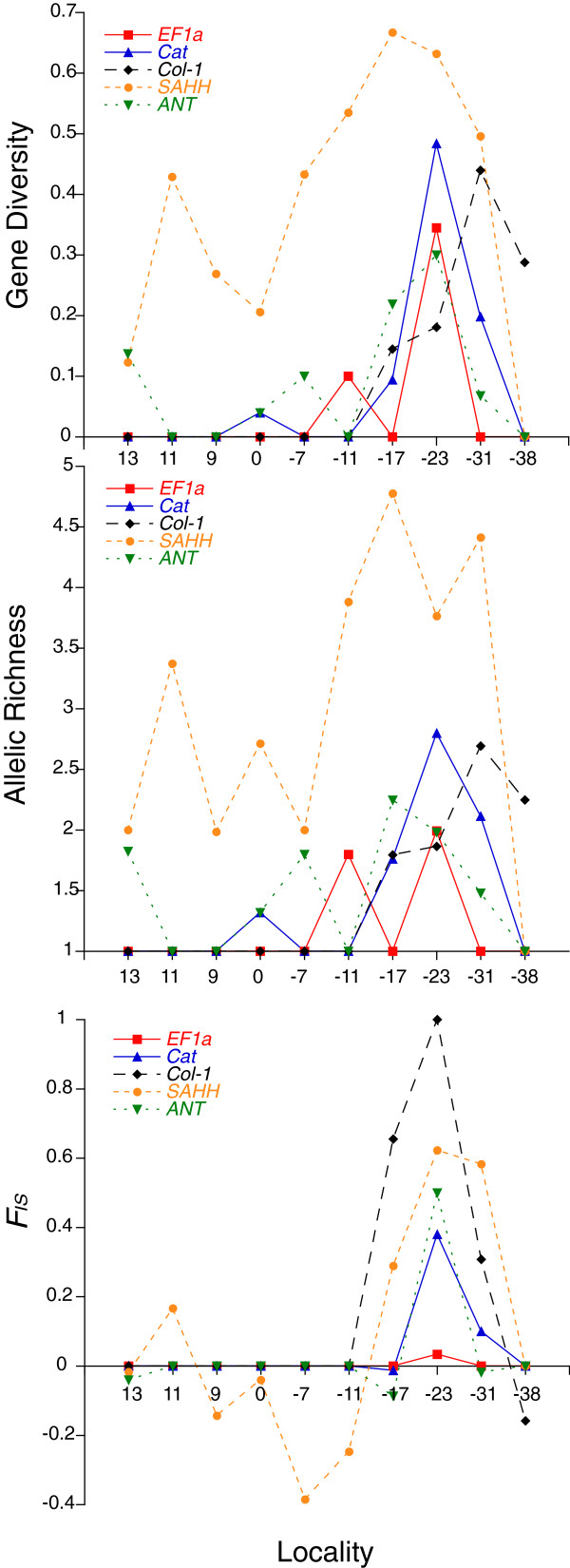
**Statistical estimates of (a) gene diversity, (b) allelic richness, and (c) heterozygote deficiencies (*F***_***IS***_**) for nuclear loci for mussel populations at each locality.**

Indices of genic diversity differed among the genes (Table 5). Diversity within localities (*h*) was greatest for *COI* and smallest for *ANT*, which was defined by one SNP, and *Col-1,* which was not polymorphic at the NEPR localities. Among the nuclear loci, *SAHH* locus had the highest diversity and richness. Expected heterozygosity and allelic richness peaked in the intergrade region, between S17 and S31, for each of the nuclear genes (Figure 4a and b). Heterozygote deficiencies (*F*_*IS*_) also peaked in the intergrade zone (Figure 4c). All statistically significant values for Fu’s *Fs* were negative (Table 5). They were most pronounced for *COI*, where nine of 13 values were significantly negative. Negative *Fs* values reflect significant excesses of rare alleles in the simple star-like nodes for *COI* (Figure 2). 

**Table 5:** **Genic diversity in eastern Pacific *Bathymodiolus *** **mussels**

**Parameter**^**1**^	**N13**	**N11**	**N9**	**GAR**	**S7**	**S11**	**S14**^**2**^	**S17**	**S18**^**2**^	**S21**^**2**^	**S23**	**S31**	**S38**	**Total**
*COI* (N)	22	21	62	48	45	21	14	135	47	34	21	41	27	538
*k*	10	12	20	21	16	8	6	37	14	20	11	17	8	121
*H*	13	17	23	28	19	11	6	34	15	20	11	23	14	96
*h*	0.810	0.871	0.753	0.856	0.839	0.776	0.769	0.841	0.710	0.934	0.819	0.870	0.658	0.890
SD	0.062	0.060	0.052	0.044	0.033	0.075	0.090	0.024	0.064	0.026	0.082	0.039	0.087	0.008
π x100	0.674	0.710	0.741	0.761	0.597	0.618	0.247	0.485	0.242	0.600	1.394	0.673	0.523	1.637
SD x100	0.393	0.412	0.420	0.425	0.353	0.366	0.186	0.292	0.174	0.357	0.754	0.383	0.314	0.094
Fu’s *Fs*	−0.625	−3.360	−6.277	−7.654	−5.578	−0.221	−2.300	−26.905	−10.272	−13.659	0.109	−5.300	−0.176	
Prob	0.369	0.044	0.023	0.003	0.012	0.500	0.021	0.000	0.000	0.000	0.554	0.031	0.508	
*SAHH* (N)	32	30	26	28	22	20	n.d.	38	n.d.	n.d.	42	46	58	232
*k*	2	6	1	3	1	6		7			3	3	3	9
*H*	3	4	2	4	2	4		6			6	5	5	14
*h*	0.123	0.402	0.271	0.206	0.485	0.553		0.617			0.623	0.707	0.525	0.806
SD	0.078	0.104	0.099	0.101	0.064	0.111		0.056			0.044	0.036	0.071	0.013
π x100	0.079	0.516	0.170	0.176	0.305	0.649		0.568			0.784	0.676	0.436	1.476
SD x100	0.138	0.423	0.213	0.217	0.305	0.504		0.449			0.493	0.504	0.372	0.894
Fu’s *Fs*	−2.437	−0.073	0.511	−2.610	1.139	0.063		−1.656			−1.977	0.004	−0.776	
Prob	0.003	0.464	0.379	0.007	0.686	0.492		0.127			0.103	0.528	0.278	
*ANT* (N)	26	28	34	52	24	22	n.d.	38	n.d.	n.d	40	54	52	370
*k*	2	1	1	2	1	1		3			2	2	1	3
*H*	1	0	0	1	0	0		1			1	1	0	1
*h*	0.148	0	0	0.039	0	0		0.240			0.296	0.073	0	0.449
SD	0.088	0	0	0.036	0	0		0.086			−0.079	0.048	0	0.020
π x100	0.029	0	0	0.008	0	0		0.048			0.060	0.015	0	0.091
SD x100	0.049	0	0	0.002	0	0		0.063			0.071	0.033	0	0.004
Fu’s *Fs*	−0.317	0	0	−1.669	0	0		−1.172			0.838	−0.949	0	
Prob	0.182	NA	NA	0.029	NA	NA		0.131			0.513	0.089	NA	
*Cat* (N)	36	26	22	50	22	20	n.d.	42	n.d.	n.d	40	56	66	380
*k*	0	0	0	1	0	0		2			2	2	0	2
*H*	1	1	1	2	1	1		3			4	3	1	4
*h*	0	0	0	0.040	0	0		0.094			0.480	0.198	0	0.465
SD	0	0	0	0.380	0	0		0.061			0.069	0.068	0	0.017
π x100	0	0	0	0.105	0	0		0.370			2.267	0.948	0	2.380
SD x100	0	0	0	0.314	0	0		0.610			1.814	1.034	0	1.834
Fu’s *Fs*	0	0	0	−1.636	0	0		−2.089			0.294	−0.222	0	
Prob	NA	NA	NA	0.040	NA	NA		0.016			0.555	0.338	NA	
*Col-1* (N)	32	28	24	24	20	22	n.d.	40	n.d.	n.d.	42	46	54	332
*k*	0	0	0	0	0	0		2			2	2	2	3
*H*	1	1	1	1	1	1		2			2	4	4	4
*h*	0	0	0	0	0	0		0.142			0.177	0.467	0.526	0.370
SD	0	0	0	0	0	0		0.071			0.074	0.070	0.071	0.034
π x100	0	0	0	0	0	0		0.274			0.340	0.777	0.715	0.652
SD x100	0	0	0	0	0	0		0.331			0.380	0.633	0.597	0.553
Fu’s *Fs*	0	0	0	0	0	0		0.773			1.159	0.252	0.167	
Prob	NA	NA	NA	NA	NA	NA		0.495			0.590	0.537	0.526	
*EF1α* (N)	32	28	22	22	18	22	n.d.	18	n.d.	n.d.	42	50	60	314
*k*	0	0	0	0	0	1		0			1	0	0	1
*H*	1	1	1	1	1	2		1			2	1	1	2
*h*	0	0	0	0	0	0.091		0			0.345	0	0	0.474
SD	0	0	0	0	0	0.081		0			0.073	0	0	0.013
π x100	0	0	0	0	0	0.505		0			1.916	0	0	2.632
SD x100	0	0	0	0	0	1.043		0			2.14	0	0	2.541
Fu’s *Fs*	0	0	0	0	0	−0.957		0			1.119	0	0	
Prob	NA	NA	NA	NA	NA	0.07		NA			0.576	NA	NA	

^1^ Abbreviations: *N* = sample size per locus; *H* = number of haplotypes; *k =* number of polymorphic sites; *h* = haplotype diversity; *π* = nucleotide diversity; SD = one standard deviation. NA = no data. Bold values indicate significance.

^2^ Data from Plouviez *et al.* 2010; n.d. indicates no data.

Except for S23, the other samples did not exhibit evidence for non-random mating. Sporadically elevated values for *F*_*IS*_ in these samples were not significant after sequential Bonferroni correction of the α-levels. Similarly, sporadic gametic disequilibria were not significant following correction for multiple tests. In contrast, violations of Hardy-Weinberg equilibrium were pronounced in the S23 sample. Three of the five nuclear loci exhibited significant heterozygote deficiencies (Table 6). Cytonuclear disequilibrium (CND) was tested in the S23 sample after recoding the mitochondrial haplotypes (Figure 2) as *N* versus *S* “mitotypes”. CND was significant for all five pairwise gametic combinations. Two of the tests remained significant following sequential Bonferroni correction (Table 6). Significant gametic disequilibrium was evident in 10 pairwise comparisons of nuclear loci. Two contrasts remained significant following sequential Bonferroni correction (Table 6).

**Table 6:** **Tests for random mating expectations in the S23 sample of eastern Pacific *Bathymodiolus *** **mussels**

	***COI***	***SAHH***	***ANT***	***CAT***	***Col-1***	***Ef1a***
*COI*	n.d.^*^					
*SAHH*	0.058^†^	***0.623***				
*ANT*	**0.004**	0.064	*0.500*			
*Cat*	**0.010**	0.439	**0.001**	***0.380***		
*Col-1*	0.015	0.213	0.053	0.053	***1.000***	
*EF1α*	0.016	0.047	**0.004**	0.073	0.035	*0.034*

^*^F_*IS*_ values for single locus genotypes are in *italics* on the diagonal (significant values in boldface; *n.d.* = not determined).

^†^*P*-values from tests for gametic disequilibrium between pairs of loci are below the diagonal. Significant test results (following sequential Bonferroni correction) are indicated in boldface.

Pairwise sequence divergence within (*d*_*w*_) and among (*d*_*a*_) sample localities was estimated separately for each locus (Additional file 1: Table S1). The mean *d*_*w*_ values were small for each locus: *COI* = 0.47%; *SAHH =* 0.46%; *ANT* = 0.01%; *Cat* = 0.38%; and *Col-1* = 0.25%. The mean *d*_*a*_ values were comparably low among localities within the northern (N13–S17) and southern (S31–S38) regions. Mean *d*_*a*_ values between samples from the different regions were typically much greater than the *d*_*w*_ values. The admixed S23 sample varied among loci in its affinities to the northern and southern regions.

**Figure 5:**
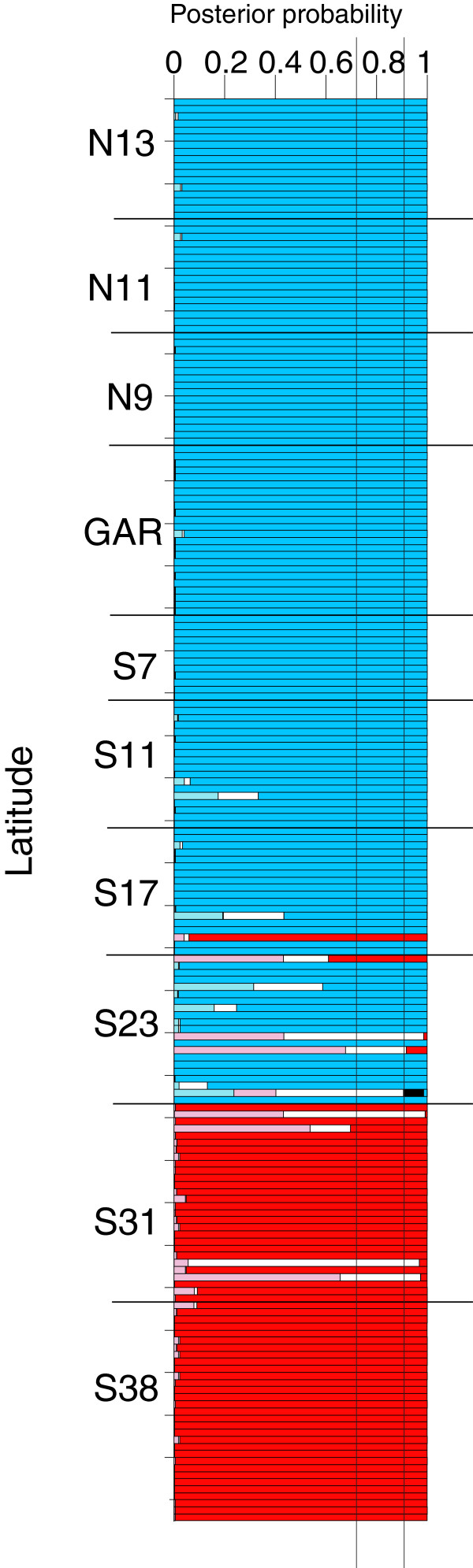
**NewHybridsanalyses of multi-locus nuclear genotypes in eastern Pacific *Bathymodiolus*.** Sample localities are ordered along the Y-axis. Vertical lines mark 0.75 and 0.90 values of posterior probabilities for assignments to the following genotypic categories: blue = northern parental; red = southern parental; black = F1 hybrid; white = F2 hybrid; light blue = northern backcross hybrid; and pink = southern backcross hybrid.

These regional differences were also revealed by multilocus genotypic assignment methods. The most probable partitioning of the total sample resulted in *K* = 3 (Additional file 1: Table S2). The northern and southern partitions were self-evident from the previous analyses. An intermediate partition composed of the S17 and S23 was influenced by the *SAHH* locus, perhaps due to recombination or incomplete lineage sorting. Exclusion of *SAHH* from the analysis resulted in an estimate of *K* = 2, with S17 and S23 nested in the northern partition. The NewHybrids analysis (Figure 5) identified four of the S23 individuals with high posterior probabilities (PP > 0.95) as hybrids, and other potential hybrids (PP < 0.75) also were noted. Due to the limited number of loci, insufficient power existed to unequivocally assign the hybrid individuals to first-generation (F1), second-generation (F2) or specific backcross categories [60]. Several putative hybrids also occurred in the S32 sample. The S17 sample had a single individual that was assigned to the southern partition (PP > 0.95).

Isolation-with-migration (IMa2) analyses were conducted with exon regions of genes that exhibited no evidence for recombination. Analyses were conducted under a two-population model, defined by the previous Structure and NewHybrid analyses, a northern (N) population pooled the N13–S23 and GAR samples, and a southern (S) population pooled the S31–S38 samples. Maximum likelihood estimates (MLEs) for the effective population size scaled by mutation rate (θ = 2*N*μ) were four-times larger for the northern population, as defined (Table 7, Additional file 2: Figure S1). Pooling would limit our interpretation of these estimates, however, if significant structuring occurred within a partition. To assess this potential problem, we excluded the S23 sample from the IMa2 analysis. This exclusion reduced the estimates of effective population size by one-half (Table 7), but θ_N_ still remained four-times greater than θ_S_. Decisions about pooling greatly influenced estimates of the immigration vectors (*M*_*i*_ = *m*_*i*_/μ). Immigration into the northern population was greater than the reverse (*M*_N_ > *M*_S_) if the S23 sample was included in the northern population. Log likelihood ratio tests on migration rates showed exclusion of S23 from the analysis resulted in no significant bidirectional migration. Estimates of ancestral population sizes (θ_A_) and population splitting time (*τ* = *tμ*) were unresolved, regardless of pooling. A sharp peak near zero in the highest posterior density (HPD) curve for *τ* is indicative of recent divergence; however, an escalating tail of the curve prevented an estimate for population splitting time from the multi-locus data.

**Table 7:** **Maximum likelihood estimates (MLE) of population parameters from IM** **a** **2 analyses from *B. thermophilus *** **cx. Lower (L) and upper (U) 95% HPD (highest posterior density) are given**

	**S23 Included**			**S23 excluded**		
**Parameter**	**MLE**	**L**	**U**	**MLE**	**L**	**U**
θ_N_	2.752	0.650	17.050	1.359	0.150	3.750
θ_S_	0.639	0.250	1.150	0.317	0	1.250
θ_A_	40.980	0.650	96.950	45.520	0	99.999
*t*	1.916	0.049	2.998	1.172	0.019	2.998
*M*_N>S_^*^	1.117†	0.285	2.475	1.579	0	4.805
*M*_S>N_^*^	0.473	0.015	1.955	3.352	0.145	8.385

^*^M_N>S_ = immigration into the north; *M*_S>N_ = immigration into the south. † Indicates significant LLR test >0.

### Recombination

We reconstructed recombination histories from the complete sequence data (with introns) for four nuclear loci. Recombination could not be tested for *ANT* because it exhibited only one polymorphic site. Coalescence analyses conducted with Beagle[45] identified large numbers of recombination events in *SAHH*, *Cat*, *Col-1* and *EF1a* (indicated with stars in Additional file 3: Figure S2). The northern and southern lineages were color-coded, as in Figure 1; however, homoplasy among the lineages obscured the identification of within- versus between-lineage recombination events. Nonetheless, the temporal distribution of recombination events suggests that the parental lineages may have had a long history of parapatric contact.

### Isolation-by-distance

Previous studies reported that *B. thermophilus* populations distributed between 13°N and 17°S exhibited evidence for isolation-by-distance [29,36]. We re-tested this hypothesis with the present mitochondrial and nuclear polymorphisms. The combined pairwise *F*_*ST*_ values for nuclear loci were not correlated with geographic distance (*r* = 0.008; *P* < 0.567, 1000 permutations) (Additional file 4: Figure S3). In contrast, *ϕ*_*ST*_ values estimated from mitochondrial sequences exhibited a significant correlation (*r* = 0.447; *P* < 0.019, 1000 permutations). Nonetheless, the *ϕ*_*ST*_ correlation resulted from contrasts involving the S17 sample. Excluding S17 from the analysis eliminated the correlation. Based on the Structure and IMa2 analyses, the S17 sample appears to be influenced by introgressed southern alleles.

### Taxonomic implications

Based on allozyme and mitochondrial DNA differences, Won *et al.*[29] suggested that the northern and southern *B. thermophilus* lineages might warrant recognition as distinct species. To assess this suggestion, we compared them with *Bathymodiolus azoricus* Cosel *et al.*[66] and *B. puteoserpentis* Cosel *et al.*[67], named geminate-species that hybridize along the Mid Atlantic Ridge. Although individuals of the MAR species can be discriminated with 95% confidence based on shell dimensions alone [24,68], the *B. thermophilus* lineages exhibit no comparable differences [29]. Also, sequence divergence for mitochondrial genes (*COI* and *ND4*) is greater between two MAR lineages, but differentiation at the nuclear loci is consistently less (Table 8). The *SAHH*, *Col-1* and *Ef1α* sequences examined in both species complexes are less between the MAR species (Table 8). Different numbers of allozyme loci were examined in the two complexes; so, we could only compare mean *F*_*ST*_ values for the subsets of polymorphic genes. The proportion of allozyme variance that exists between the MAR species is half that of the *B. thermophilus* lineages. To summarize, shell dimensions and mitochondrial sequences indicate that the MAR species are more divergent than *B. thermophilus* lineages, but the opposite is true for nuclear genes. Because adaptively neutral divergence in protein-coding nuclear genes is generally believed to accumulate much more slowly than in mitochondrial genes, the consensus of evidence suggests that the EPR lineages split earlier than the MAR species.

**Table 8:** **Genetic divergence between northern and southern lineages of *B. thermophilus *** **and *B. antarcticus *** **(N/S), and between *B. azoricus *** **and *B puteoserpentis *** **(*az/pu*) from the Mid-Atlantic Ridge**

**Marker**	**Term**	**N/S**	***az/pu***	**Refs.**^**1**^
*COI*	*d*	0.046	0.058	1, 3
*ND4*	*d*	0.063	0.130	4, 4
*SAHH*	*d*	0.020	0.008	5, 6
*Col1*	*d*	0.008	0	5, 6
*EF1α*	*d*	0.008	0	5, 6
Allozymes	*F*_*ST*_	0.480	0.240	1, 2

^1^ References: 1. [24]; 2. [68]; 3. [16]; 4. [69]; 5. this study; 6. [25].

Assessing the species-status of allopatric and parapatric populations is an old problem in biology. Here we adopt the “metapopulation species concept” which equates species with separately evolving segments of the broader metapopulation [70]. In this sense, the widely distributed metapopulation known as *B. thermophilus* sensu latu is clearly partitioned into separately evolving metapopulation segments. Mixed nuclear genotypes in the hybrid zone reveal that northern and southern lineages can still hybridize. Yet, the capability to hybridize does not prove they are conspecific, as many recognized plant and animal species hybridize at contact zones or in disturbed environments [5]. Consequently, we hereby elevate the southern lineage to species status, and restrict the application of *B. thermophilus* sensu stricto to populations occurring to the north of the Easter Microplate hybrid zone.

SYSTEMATICS

Family MYTILIDAE Rafinesque, 1815

Subfamily Bathymodiolinae Kenk & Wilson, 1985

Type genus *Bathymodiolus* Kenk & Wilson, 1985

*Bathymodiolus antarcticus* Johnson & Vrijenhoek new species

(Figure 6)

**Figure 6:**
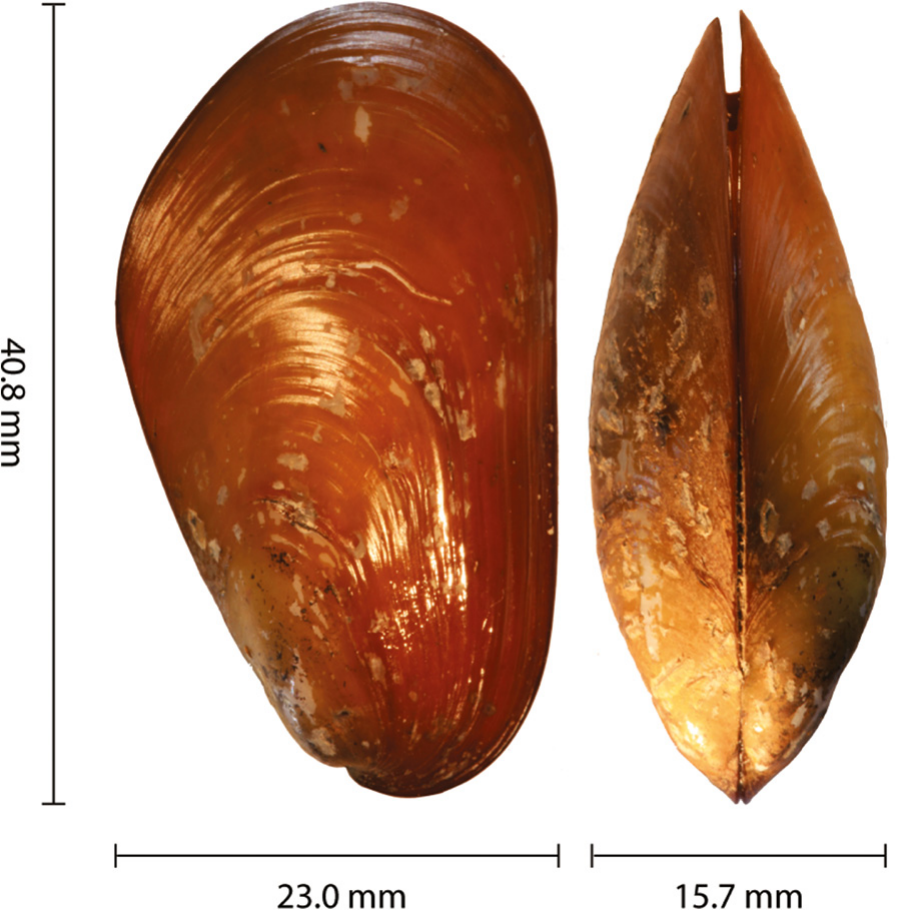
***Bathymodiolus antarcticus *n. sp. paratype (USNM: 1196520).A.** lateral view of right valve. **B.** Posterior view of hinge, left and right valves.

*Bathymodiolus* aff. *thermophilus*[29,37,71-73]*Bathymodiolus* cf. *thermophilus*[74]

Type material. — Holotype: sampled at 37.78°S/110.90°W on the Pacific-Antarctic Ridge, 2200 meters depth during *Alvin* dive A4088 (Table 1). Preserved in 10% sea-water buffered formalin, then transferred to ethanol (USNM 1196518). Paratypes: sampled at 31°51S/112°W, on the East Pacific Rise, 2336 meters depth during *Alvin* dive A4092 (Table 1). Preserved in ethanol (6 USNM 1196519; 6 SMNH 8414–8415). Species registered with Zoobank: urn:lsid:zoobank.org:act:47DBB0D0-E4A8-49DE-9EC5-A86133CFBACA.

Diagnosis. — This species differs from *B. thermophilus* s.s. by the following combination of character states of its mitochondrial *COI* barcode: 126C, 160A, 217C, 276 T, 282 G/A, 330 T, 390A, 403 T, 504 T, 519 T, 547C, 558C, 570A, and 603C. It also differs diagnostically for exon regions the *SAHH* gene. Morphological differences are unknown.

Distribution. — Known from hydrothermal vent localities between 31°09^′^S on the East Pacific Rise to 37°47^′^S on the Pacific-Antarctic Ridge.

Etymology. — Latin adjective *antarcticus* (ant’arc.ti.cus), meaning opposite the North Star, and referring to its distribution on the Pacific-Antarctic Ridge.

*Bathymodiolus thermophilus* sensu stricto, Kenk & Wilson, 1985, as described

Type material. — Holotype: USNM 803661, preserved in 70% ethanol. Collected 20 January 1979 by Ellis and Ballard on Alvin dive 879 from Mussel Bed (0⋅ 47.89’N; 86⋅ 9.21’W) at 2495 m depth on the Galápagos Rift.

Revised diagnosis. — This species differs from *B. antarcticus* n. sp. by the following combination of character states of its mitochondrial *COI* barcode: 126 T, 160 G, 217 T, 276C, 282 T, 330C, 390C, 403C, 504 G/A, 519C/A, 547 T, 558 T, 570 G, and Type material. — Holotype: United States National Museum (USNM 803661), preserved in 70% ethanol. Collected 20 January 1979 by Ellis and Ballard on *Alvin* dive 879.

Distribution. — Known from hydrothermal vents on the Galápagos Rift between 0.80°N/86.15 and 0.80°N/86.122 W, and from the East Pacific Rise (EPR) between 12.80°N/103.93°W and 21.57°S/114.4 W.

## Discussion

This is the second report of a mid-ocean hybrid zone involving hydrothermal vent mussels [16,29]. As in the previous case involving the Mid-Atlantic Ridge (MAR) mussels, the present example from the eastern Pacific Ocean also exhibits the properties of a Tension Zone: (1) coincident clines at multiple loci; (2) heterozygote deficiencies in the contact zone; and (3) significant gametic disequilibrium among nuclear and cytoplasmic genes (Figures 2, 3, 4, Table 6). Tension Zones are maintained by hybrid unfitness (i.e. endogenous selection) and recurrent immigration from the parental regions [1,75,76]. The abrupt step-clines at six independent gene loci suggest that introgression has been limited to adjacent localities.

We could not ascertain from the present genetic data whether the excess proportions of parental genotypes in the hybrid zone were products of selection against hybrids, or consequences of admixture (i.e. Wahlund effects). Comparison of juvenile and adult cohorts might help to identify ongoing selection against hybrids e.g., [77], but we are not aware of future efforts to explore this remote region with submersible vehicles. Simple admixture of immigrant recruits from the adjacent parental regions would generate the observed heterozygote deficiencies, gametic phase disequilibrium [78], and putative hybrid genotypes in the S23 sample (Figure 5). Although the S23 vent field appeared to be in a senescent stage of the hydrothermal cycle a single active vent chimney supported small populations of chemosynthetically dependent animals. We observed one living adult mussel (unsampled) while reviewing the dive videos from 14 hrs of *Alvin* ‘bottom-time’. Shell debris from recently deceased bivalves littered the floor of the crevice that contained the active chimney. Other vents hosting more robust mussel populations might exist nearby, but they remain undiscovered.

“Sweepstakes Reproductive Success” (SRS) as observed in some near-shore bivalves and other organisms reviewed in [42], might be invoked to explain the heterozygote deficiencies, but the hybrid zone juveniles were not the progeny of few females. The sample of 21 juveniles contained 11 distinct mitochondrial haplotypes (Table 5). Haplotypic diversity (*h* = 0.819) was not different from the mean diversity (*h* = 0.807 ± 0.077) for all other samples, which exhibited no evidence for deviations from random-mating expectations. The planktotrophic larvae of *Bathymodiolus* mussels appear to mix thoroughly before they settle at vents. Their capacity for long-distance dispersal is consistent with a lack of geographical subdivision among EPR (N13–S17) and GAR populations spanning thousands of kilometers [79] and this study. A previous report of subdivision across the Equator is an artifact of sampling gaps cf. [21,36]. Nonetheless, broad-scale homogeneity could coincide with small-scale patchiness in genotypic frequencies under the SRS model [42]. Hypervariable genetic markers might reveal localized patchiness due to episodic recruitment of cohorts from a limited number of parents in different source populations. For example, AFLP variation provides preliminary evidence for small-scale patchiness in the eastern Pacific vent annelid *Riftia pachyptila*[80], despite broad-scale homogeneity of nuclear and mitochondrial DNA markers [31]. In contrast, microsatellite DNAs provided no evidence for small-scale patchiness in the western Pacific vent snail, *Ifremeria nautileii*[81].

### Divergence and intergradation

Reciprocally monophyletic differences for mitochondrial haplotypes and step-clines in allozyme frequencies suggest a relatively long history of isolation between *B. antarcticus* n. sp. and *B. thermophilus s.s*. Based on borrowed molecular clock rates for *COI* (1–2% divergence per million years), we estimated that the northern and southern mitochondrial lineages split 2.1– 4.3 Myr ago, which temporally overlaps a span of dates encompassing orogeny of the Easter Microplate, 2.5–5.3 Myr ago [82,83]. An ongoing study that uses fossil data to calibrate major splitting events in the subfamily Bathymodiolinae estimates a similar time for the most recent common ancestor of the mitochondrial lineages (J. Lorien, personal communication). Nonetheless, mitochondrial ‘gene trees’ do not necessarily equal ‘species trees’ [84]. Different mutation rates among nuclear and mitochondrial genes, the amounts of ancestral polymorphism in various genes, and differing intensities of selection on independent characters can greatly complicate relationships between gene and organismic trees. Disagreements between these trees are further complicated by introgressive hybridization and reticulation [85]. Thus, it is not surprising that IMa2 analysis failed to resolve a splitting time (*τ*) from the multi-locus data for these mussels.

Whether intergradation between the northern and southern lineages results from primary or secondary processes remains uncertain. A coalescent analysis of the MAR mussel species provided evidence for “an initial period of allopatric differentiation during which recombination was blocked between lineages” followed by “a long history of gene flow” [25]. In contrast, our analysis of past recombination in the eastern Pacific mussels is consistent with a long history of parapatric contact. However, these contacts probably are episodic in this dynamic region of the global ridge system. Chaotic local extinctions and colonizations of vents as tectonic activities move up and down a ridge axis can create transient contact zones that allow gene flow [86]. The observed recombination history of these mussel lineages is consistent with episodic isolation and re-contact as tectonic conditions vary temporally.

### Isolating mechanisms

Density troughs (regions of low population size) can function as powerful traps to stabilize hybrid zones and limit introgression [87-89]. The MAR hybrid zone coincides with a density trough at ‘Broken Spur’ [16,24], a geographically intermediate vent field that is relatively inhospitable for mussels due to precipitation of toxic polymetalic sulfides believed to interfere with respiration [23]. Mussel population densities were also low in the Easter microplate region during our 1999 and 2005 expeditions, but the causes are different. First, Won *et al.*[29] hypothesized that strong cross-axis currents will remove planktotrophic mussel larvae from the ridge axis, limiting local self-recruitment. These superfast-spreading centers have highly inflated axial calderas and essentially no lateral walls to constrain axial circulation [90]. The predominantly cross-axis currents in this region drive buoyant hydrothermal plumes to the west [91] presumably carrying larvae that rise above the axial calderas. Secondly, Coykendall *et al.*[31] hypothesized that vent habitats along the southern EPR are subject to high rates of disturbance. The habitats are frequently “repaved” with lava flows that drive local extinctions and create new vents [74]. We explored a number of hydrothermally active vent fields between 18.40°and 21.73°S latitude during our January 1999 expedition (Table 1), but none of them supported robust mussel populations. Soon after, Van Dover [92] visited the ‘Animal Farm’ vent field at 18.60°S and found it “clearly in a waning stage of the hydrothermal cycle’ (p. 142). Venting water temperatures were only slightly elevated above ambient and most of the mussels were dead. The living individuals were smaller on average compared to dense mussel populations just to the north (Table 1). The BIOSPEEDO expedition in 2004 sampled mussels at 18.40°and 21.42°S, but population densities were not reported [36]. Demographic fluctuations result in variance-effective population sizes that might be very small for some taxa, leading to losses of genetic variation [22]. For example, southern EPR populations of *Riftia pachyptila* are nearly devoid of nuclear and mitochondrial sequence variation [31], but other annelids are not similarly affected [30,36]. Together, cross-axis currents and metapopulation processes might reduce the genetically effective population size of mussel populations in this region, impeding along-axis dispersal and limiting introgression across the Easter Microplate.

## Conclusions

Although *B. thermophilus* s.s. and *B. antarcticus* n. sp. cannot be distinguished with shell measurements, they differ in having reciprocally monophyletic mitochondrial lineages. Polymorphisms at nuclear loci (allozymes and DNA sequences) are not completely diagnostic but the two species comprise discrete genotypic clusters with predominantly non-overlapping geographical distributions. Some shared alleles might be remnants of incomplete lineage sorting between the metapopulation lineages, but these alleles are not widely distributed geographically, as expected for ancestral polymorphisms. Instead, they only penetrate localities that flank the Easter Microplate, a pattern expected for introgressive hybridization or disruptive selection along an environmental gradient [1].

Divergence between the two species probably initiated with orogeny of the Easter Microplate, 2.5–5.3 Myr ago. Superfast tectonic spreading rates in this dynamic part of the global mid-ocean ridge system result in frequent habitat destruction, leaving episodic gaps in the distribution of many vent-restricted species. Prolonged periods of partial to complete isolation would facilitate the accumulation of genetic differences in nuclear and mitochondrial genes exposed to genetic drift and potentially contrasting selection regimes. We suggest two hypotheses about possible adaptions that might result in disruptive selection across the Easter Microplate boundary. First, we hypothesize that novel life history traits emerged to accommodate different current regimes in the northern and southern regions. Northern *B. thermophilus* s.s. populations are subjected to a circulation pattern dominated by mesoscale eddies and along-axis currents [20,93-95]. There, larval buoyancy and duration probably evolved to favor the retention of the planktotrophic prodissoconch stages. South of the Easter Microplate, *B. antarcticus* n. sp. is subjected to strong cross-axis vectors forced by the Antarctic Circumpolar Current [29]. Larval buoyancies and durations that function in the northern current regime would result in larval removal in this contrasting regime [96]. Though we currently have no developmental data on the larval life histories of these mussels, this hypothesis is testable. Early settling post-larvae retain their developmental history in prodissoconch sizes and morphologies that can provide significant clues regarding larval life history [97].

Secondly, we hypothesize that coadaptation between the mussel hosts and associated thiotrophic endosymbionts might result in disruptive selection. *B. thermophilus* s.s. and *B. antarcticus* n. sp. differ with respect to the symbiont strains they host in specialized cells (bacteriocytes) contained mostly in gill tissues [98]. Though we cannot at this time exclude some degree of vertical transmission, the endosymbionts are mostly acquired locally from the environment in which mussel larval settle [99]. An ongoing multi-gene analysis of symbiont diversity has only found the *B. thermophilus* s.s. symbiont strain in the hybrid zone mussels (Y-J Won, unpublished data), even in host individuals with predominantly southern genotypes. Subtle interactions involving these intracellular microbes might have affected evolution in mitochondrial and nuclear genes of the hosts. According to the “Red King” model, infectious horizontal symbionts and their hosts are expected to exhibit decelerated evolutionary rates, because the participants are subject to constraints that limit any form of change [100,101]. Episodic range expansions into the contact zone will result in secondary contacts that re-establish opportunities for gene flow and introgression in the hosts, and possible symbiont transfers. Yet, conflicts between divergent larval strategies, symbiont coadaptation, and other unknown processes would determine the strength of selection against intergrades. Disruptive processes involving the mussel hosts and their obligate endosymbionts might contribute to partial isolation and incipient speciation of these mussels.

## Competing interest

The authors declare that they have no competing interest.

## Author’s contributions

SBJ conducted most of the DNA sequencing and statistical analyses with the assistance of YJW and JBH. RCV led oceanographic expeditions that with the help of SBJ and YJW collected mussel specimens. SBJ, RCV and YJW drafted the manuscript. All authors read and approved the final manuscript.

## Supplementary Material

Additional file 1 **Table S1.** Mean percentage of sequence divergence (K2P corrected) within (*d*_*w*_, boldface on diagonal) and between (*d*_*a*_ off-diagonal) eastern Pacific *Bathymodiolus* mussels. Pairwise comparisons involving the S23 sample are highlighted in blue. The *d* values between northern and southern regions are highlighted in gray. **Table S2.** Simulation results for the Structure analysis of eastern Pacific *Bathymodiolus* mussels under correlated and uncorrelated allelic frequency models. The number of possible genotypic clusters, *K*, varied from one to eleven. The natural log of the probability of the data for a given value of *K* was averaged across the number of simulations (runs) per *K*. Bayes factors were estimated based on these averages. Click here for file

Additional file 2 **Figure S1.** IMA2 posterior distributions for demographic parameters in *B. thermophilus* under a two-population model: a) N13–S23 vs. S31–S38; b) N13–N21 vs. S31–S38. Click here for file

Additional file 3 **Figure S2.** Recombination analyses for the loci (a) *EF1α,* 12 recombination events, (b) *Col-1,* 17 recombination events, (c) *SAHH,* 11 recombination events, and (d) *Cat,* 10 recombination events*.* Open circles represent mutations and stars represent recombination events. Colors on OTUs are blue (NEPR), green (GAR), yellow (SEPR), and red (PAR). Click here for file

Additional file 4 **Figure S3.** Analysis of isolation-by-distance in *B. thermophilus* populations from north of the Easter Microplate. Open triangles are *φ*_*ST*_ values estimated from mitochondrial sequences in the GAR and EPR (N13–S21) samples. Filled circles are *F*_*ST*_ values estimated from the combined nuclear loci in the GAR, and EPR (N13–S17) samples. Click here for file
