# Supplementary material for: A hybrid zone between Bathymodiolus mussel lineages from eastern Pacific hydrothermal vents
Source: BMC Evol Biol. 2013 Jan 24;13:21. doi: 10.1186/1471-2148-13-21 (PMC3740784; doi:10.1186/1471-2148-13-21)
Supplement: Additional file 1 — Table S1. Mean percentage of sequence divergence (K2P corrected) within (dw, boldface on diagonal) and between (da off-diagonal) eastern Pacific Bathymodiolus mussels. Pairwise comparisons involving the S23 sample are highlighted in blue. The d values between northern and southern regions are highlighted in gray. Table S2. Simulation results for the Structure analysis of eastern Pacific Bathymodiolus mussels under correlated and uncorrelated allelic frequency models. The number of possible genotypic clusters, K, varied from one to eleven. The natural log of the probability of the data for a given value of K was averaged across the number of simulations (runs) per K. Bayes factors were estimated based on these averages. [file 1471-2148-13-21-S1.docx]

**Supplementary Table S1.** Mean percentage of sequence divergence (K2P corrected) within (*d_w_*, boldface on diagonal) and between (*d_a_* off-diagonal) eastern Pacific *Bathymodiolus* mussels. Pairwise comparisons involving the S23 sample are highlighted in blue. The *d* values between northern and southern regions are highlighted in gray.

| Locus | Locality: | N13 | N11 | N9 | GAR | S7 | S11 | S17 | S23 | S31 | S38 |  |
| --- | --- | --- | --- | --- | --- | --- | --- | --- | --- | --- | --- | --- |
| *COI* | N13 | **0.328** |  |  |  |  |  |  |  |  |  |  |
|  | N11 | 0.320 | **0.321** |  |  |  |  |  |  |  |  |  |
|  | N9 | 0.342 | 0.343 | **0.372** |  |  |  |  |  |  |  |  |
|  | GAR | 0.343 | 0.345 | 0.366 | **0.374** |  |  |  |  |  |  |  |
|  | S7 | 0.275 | 0.278 | 0.300 | 0.301 | **0.246** |  |  |  |  |  |  |
|  | S11 | 0.340 | 0.337 | 0.366 | 0.368 | 0.300 | **0.373** |  |  |  |  |  |
|  | S17 | 0.288 | 0.234 | 0.311 | 0.313 | 0.246 | 0.279 | **0.022** |  |  |  |  |
|  | S23 | 0.984 | 0.938 | 1.009 | 1.010 | 0.941 | 0.979 | 0.754 | **1.345** |  |  |  |
|  | S31 | 5.106 | 5.105 | 5.134 | 5.133 | 5.059 | 5.131 | 5.072 | 4.449 | **0.804** |  |  |
|  | S38 | 4.962 | 4.961 | 4.990 | 4.990 | 4.915 | 4.987 | 4.928 | 4.306 | 0.682 | **0.568** |  |
| *SAHH* | N13 | **0** |  |  |  |  |  |  |  |  |  | |
|  | N11 | 0.478 | **0.884** |  |  |  |  |  |  |  |  | |
|  | N9 | 0.158 | 0.578 | **0.253** |  |  |  |  |  |  |  | |
|  | GAR | 0.119 | 0.563 | 0.238 | **0.227** |  |  |  |  |  |  | |
|  | S7 | 0.226 | 0.621 | 0.271 | 0.288 | **0.313** |  |  |  |  |  | |
|  | S11 | 0.409 | 0.770 | 0.454 | 0.472 | 0.474 | **0.679** |  |  |  |  | |
|  | S17 | 2.309 | 2.230 | 2.463 | 2.427 | 2.530 | 2.443 | **0.600** |  |  |  | |
|  | S23 | 2.313 | 2.241 | 2.477 | 2.436 | 2.547 | 2.450 | 0.544 | **0.498** |  |  | |
|  | S31 | 2.463 | 2.469 | 2.627 | 2.586 | 2.697 | 2.559 | 1.021 | 0.896 | **0.709** |  | |
|  | S38 | 2.155 | 2.158 | 2.318 | 2.277 | 2.388 | 2.276 | 0.719 | 0.598 | 0.610 | **0.401** | |
| *ANT* | N13 | **0** |  |  |  |  |  |  |  |  |  | |
|  | N11 | 0 | **0** |  |  |  |  |  |  |  |  | |
|  | N9 | 0 | 0 | **0** |  |  |  |  |  |  |  | |
|  | GAR | 0 | 0 | 0 | **0** |  |  |  |  |  |  | |
|  | S7 | 0 | 0 | 0 | 0 | **0** |  |  |  |  |  | |
|  | S11 | 0 | 0 | 0 | 0 | 0 | **0** |  |  |  |  | |
|  | S17 | 0.011 | 0.011 | 0.011 | 0.011 | 0.011 | 0.011 | **0.022** |  |  |  | |
|  | S23 | 0.035 | 0.035 | 0.035 | 0.035 | 0.035 | 0.035 | 0.043 | **0.060** |  |  | |
|  | S31 | 0.197 | 0.197 | 0.197 | 0.197 | 0.197 | 0.197 | 0.186 | 0.163 | **0.010** |  | |
|  | S38 | 0.202 | 0.202 | 0.202 | 0.202 | 0.202 | 0.202 | 0.191 | 0.167 | 0.005 | **0** | |
| *Cat* | N13 | **0** |  |  |  |  |  |  |  |  |  | |
|  | N11 | 0 | **0** |  |  |  |  |  |  |  |  | |
|  | N9 | 0 | 0 | **0** |  |  |  |  |  |  |  | |
|  | GAR | 0.054 | 0.054 | 0.054 | **0.107** |  |  |  |  |  |  | |
|  | S7 | 0 | 0 | 0 | 0.054 | **0** |  |  |  |  |  | |
|  | S11 | 0 | 0 | 0 | 0.054 | 0 | **0** |  |  |  |  | |
|  | S17 | 0.195 | 0.195 | 0.195 | 0.243 | 0.195 | 0 | **0.383** |  |  |  | |
|  | S23 | 1.642 | 1.642 | 1.642 | 1.663 | 1.642 | 1.642 | 1.719 | **2.357** |  |  | |
|  | S31 | 4.944 | 4.944 | 4.944 | 4.898 | 4.944 | 4.944 | 4.785 | 3.620 | **0.986** |  | |
|  | S38 | 5.484 | 5.484 | 5.484 | 5.428 | 5.484 | 5.484 | 5.287 | 3.836 | 0.538 | **0** | |
| *Col-1* | N13 | **0** |  |  |  |  |  |  |  |  |  | |
|  | N11 | 0 | **0** |  |  |  |  |  |  |  |  | |
|  | N9 | 0 | 0 | **0** |  |  |  |  |  |  |  | |
|  | GAR | 0 | 0 | 0 | **0** |  |  |  |  |  |  | |
|  | S7 | 0 | 0 | 0 | 0 | **0** |  |  |  |  |  | |
|  | S11 | 0 | 0 | 0 | 0 | 0 | **0** |  |  |  |  | |
|  | S17 | 0.147 | 0.147 | 0.147 | 0.147 | 0.147 | 0.147 | **0.276** |  |  |  | |
|  | S23 | 0.187 | 0.187 | 0.187 | 0.187 | 0.187 | 0.187 | 0.305 | **0.346** |  |  | |
|  | S31 | 1.388 | 1.388 | 1.388 | 1.388 | 1.388 | 1.388 | 1.331 | 1.314 | **0.882** |  | |
|  | S38 | 1.041 | 1.041 | 1.041 | 1.041 | 1.041 | 1.041 | 1.035 | 1.030 | 0.974 | **1.037** | |

**Supplementary Table S2.** Simulation results for the Structure analysis of eastern Pacific *Bathymodiolus* mussels under correlated and uncorrelated allelic frequency models. The number of possible genotypic clusters, *K*, varied from one to eleven. The natural log of the probability of the data for a given value of *K* was averaged across the number of simulations (runs) per *K*. Bayes factors were estimated based on these averages.

|  | Correlated allele frequencies | | |  | Uncorrelated allele frequencies | | |
| --- | --- | --- | --- | --- | --- | --- | --- |
| K | Bayes  factor | Mean ln Pr(X\|K) | runs |  | Bayes factor | Mean ln Pr(X\|K) | runs |
| 1 | 0.0000 | -979.42 | 11 |  | 0.0000 | -979.50 | 10 |
| 2 | 0.0000 | -508.40 | 11 |  | 0.0000 | -535.46 | 10 |
| 3 | 1.0000 | -413.63 | 11 |  | 1.0000 | -459.11 | 10 |
| 4 | 0.0000 | -438.54 | 11 |  | 0.0000 | -471.64 | 10 |
| 5 | 0.0000 | -473.85 | 11 |  | 0.0000 | -485.62 | 10 |
| 6 | 0.0000 | -465.53 | 11 |  | 0.0000 | -503.38 | 10 |
| 7 | 0.0000 | -457.70 | 11 |  | 0.0000 | -517.81 | 10 |
| 8 | 0.0000 | -448.50 | 11 |  | 0.0000 | -542.89 | 10 |
| 9 | 0.0000 | -566.60 | 11 |  | 0.0000 | -567.88 | 10 |
| 10 | 0.0000 | -502.50 | 11 |  | 0.0000 | -590.02 | 10 |
| 11 | 0.0000 | -979.42 | 11 |  | 0.0000 | -979.50 | 10 |
